# Afforestation neutralizes soil pH

**DOI:** 10.1038/s41467-018-02970-1

**Published:** 2018-02-06

**Authors:** Songbai Hong, Shilong Piao, Anping Chen, Yongwen Liu, Lingli Liu, Shushi Peng, Jordi Sardans, Yan Sun, Josep Peñuelas, Hui Zeng

**Affiliations:** 1 Sino-French Institute for Earth System Science, College of Urban and Environmental Sciences, Peking University, 100871 Beijing, China; 2 Key Laboratory of Alpine Ecology and Biodiversity, Institute of Tibetan Plateau Research, Center for Excellence in Tibetan Earth Science, Chinese Academy of Sciences, 100085 Beijing, China; 3 Department of Forestry and Natural Resources, Purdue University, West Lafayette, IN 46907 USA; 4 State Key Laboratory of Vegetation and Environmental Change, Institute of Botany, Chinese Academy of Sciences, 100093 Beijing, China; 5 CREAF, Cerdanyola del Vallès, 08193 Barcelona, Catalonia Spain; 6 CSIC, Global Ecology Unit CREAF-CSIC-UAB, Bellaterra, 08193 Barcelona, Catalonia Spain; 7 Peking University Shenzhen Graduate School, 518055 Shenzhen, China

## Abstract

Soil pH regulates soil biogeochemical processes and has cascading effects on terrestrial ecosystem structure and functions. Afforestation has been widely adopted to increase terrestrial carbon sequestration and enhance water and soil preservation. However, the effect of afforestation on soil pH is still poorly understood and inconclusive. Here we investigate the afforestation-caused soil pH changes with pairwise samplings from 549 afforested and 148 control plots in northern China. We find significant soil pH neutralization by afforestation—afforestation lowers pH in relatively alkaline soil but raises pH in relatively acid soil. The soil pH thresholds (*T*_pH_), the point when afforestation changes from increasing to decreasing soil pH, are species-specific, ranging from 5.5 (*Pinus koraiensis*) to 7.3 (*Populus* spp.) with a mean of 6.3. These findings indicate that afforestation can modify soil pH if tree species and initial pH are properly matched, which may potentially improve soil fertility and promote ecosystem productivity.

## Abstract (web summary)

Afforestation is often used to increase terrestrial carbon sequestration and restore ecosystem services. Here, the authors show that afforestation can also neutralize soil pH by lowering pH in alkaline soil but raising pH in acid soil, thus further promoting the restoration of ecosystem functions.

## Introduction

Soil pH, which measures the acidity or alkalinity of soils, is associated with many soil properties such as hydrolysis equilibrium of ions^1^, microbial communities^2,3^, and organic matter contents^4^. Recent climate and anthropogenic changes have significantly modified soil properties including soil pH^5,6^. In particular, soil acidification has been widely reported across a variety of ecosystem types and regions^5–7^. Change in soil pH may alter soil biogeochemical processes, and has cascading effects on terrestrial ecosystem structure and functions^8–12^. For instance, soil acidification caused by nitrogen deposition has led to diversity loss throughout the world^8,9^. Further soil acidification could increase leaching loss of cation nutrients, intensifying the scarcity of some nutrient elements essential for plant growth, thus reducing plant productivity^10^. Accordingly, the impacts of soil acidification have attracted increasing concerns from both the scientific community and the public and raised discussions on possible mitigation measures^13^, and it is generally agreed that the potential impact on soil pH needs to be included in the design and evaluation of many land use change projects^14,15^.

Afforestation is one increasingly popular type of land use change projects primarily designated for wood production, soil and water conservation, increasing carbon storage and mitigating climate change^16^. However, it could also change soil pH^14,15,17,18^. Soil pH is determined by the balance between production and consumption of soil hydrogen ions^19,20^, which is closely associated with nutrient (e.g. carbon, nitrogen, phosphorus, sulfur, calcium) cycles^19–23^. Afforestation could affect the nutrient cycles through plant uptake of exchangeable cations^17,23^, capture of acid deposition^19,20^, modification in the quality, and quantity of litter input and rhizosphere processes^19,20^, which consequently impact the generation and consumption of soil hydrogen ions and soil pH. It is commonly reported that afforestation decreases soil pH, although results widely varied among different studies and different regions^17,18,23,24^. Furthermore, limited by research scale, we still know little on the spatial heterogeneity of afforestation-induced soil pH changes, and on how factors like background soil physical and chemical properties, and species choices may influence soil pH. Large-scale comparative studies between afforested and non-afforested control sites may provide critical information to address these questions.

**Fig. 1:**
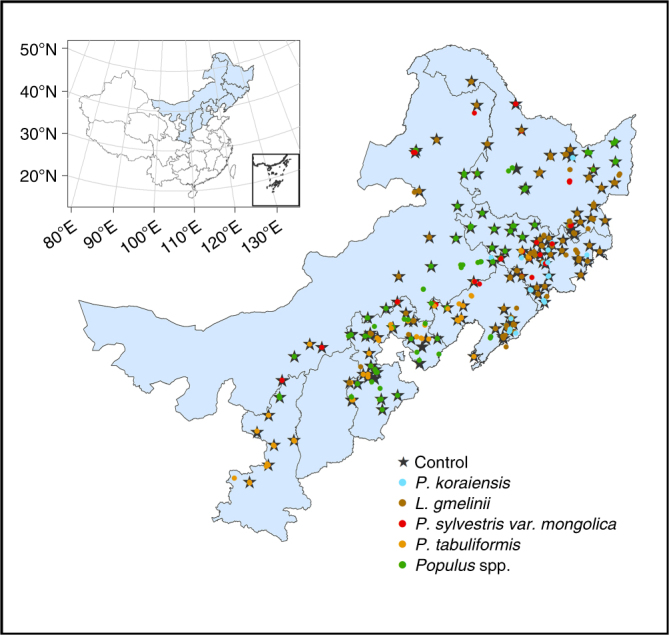
Location of the study region in China and the study plots. The pentagrams represent the control plots and the cycles of different colors represent different planted tree species at each plot. The inset shows the location of the study region in China. This map was created in ArcGIS 10.1

The primary objective of this study is therefore to investigate the effect of afforestation on soil pH across different tree species and soil pH gradient. To achieve this goal, we conducted a comparative study using samples from northern China, a broad geographical region spanning 2000 km from east to west and known for ambitious state-sponsored large-scale afforestation efforts. China is the world’s largest cultivator of forest plantations, and afforestation has contributed to approximately 90% of its forest area expansion and 49.3% of its forest carbon sink since the 1970s^25^. The Three-North Shelterbelt Development Program (TNSDP), lying in North, Northwest, and Northeast China and covering an area of 4,069,000 km^2^ since 1978^26^, is one of China’s earliest large-scale afforestation projects. The TNSDP program has generated important environmental and socioeconomic benefits, including reduced soil erosion and sand storms, and increased carbon sequestration^27,28^. This large-scale afforestation project also provides a rare opportunity to investigate the effects of afforestation on soil properties. In 2012−2013, we sampled across 148 sites within the TNSDP area to conduct pairwise comparisons on soil pH between planted forests and non-afforested control plots. For each site, we sampled one non-afforested control plot and several afforestation plots (see the section “Methods”) of different stand ages, resulting in a sum of 697 sampled plots (Fig. 1, 549 afforested and 148 non-afforested control plots) that made 549 afforestation−control pairs (note most of the control plots (119 of 148) corresponded to more than one afforestation plots). Five tree species, *Pinus (P.) koraiensis*, *Larix (L.) gmelinii*, *Pinus (P.) sylvestris* var. *mongolica*, *Pinus (P.) tabuliformis* and *Populus* spp., were used in these afforestation plots; and all plots were monocultures. Our results show significant soil pH neutralization by afforestation—afforestation decreases soil pH in alkaline soils but increases soil pH in acidic soils. These findings provide improved understandings on how afforestation impacts soil pH across a broad range of soil types and afforestation tree species, which is critical for developing climate change mitigation strategies and ecological sustainability plans.

## Results

### The overall effect of afforestation on soil pH

**Fig. 2:**
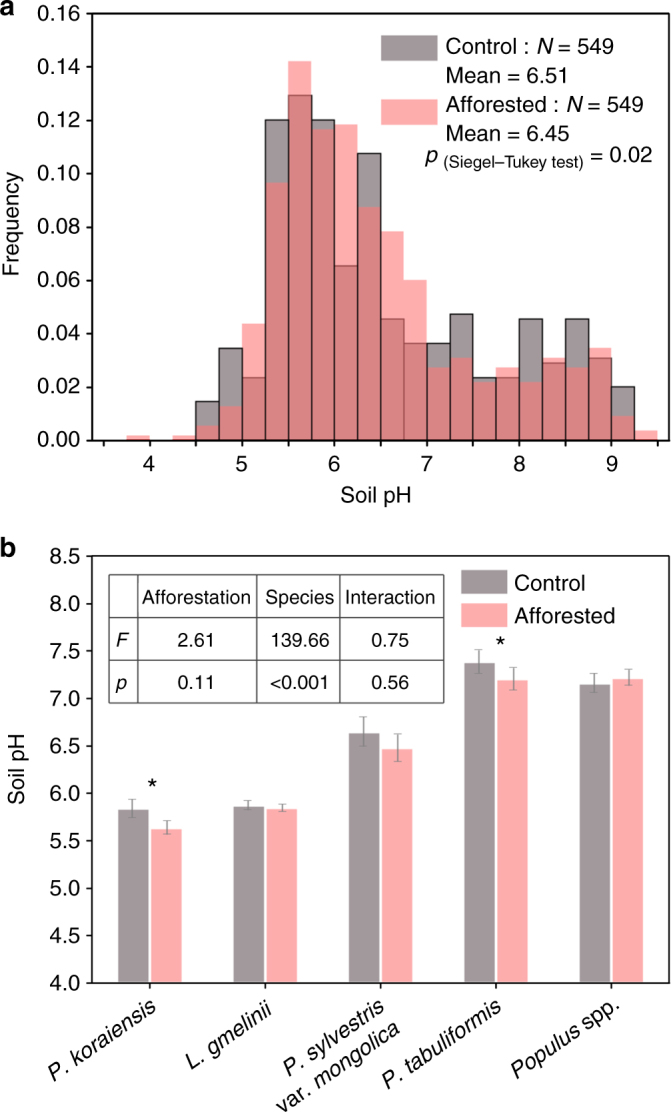
Comparison of soil pH in control and afforested groups. **a** The frequency distribution of soil pH in control and afforested groups. Siegel−Tukey test was used to compare the frequency distributions of soil pH between the control and afforested groups. **b** Soil pH for the control and afforested groups across five tree species. Error bars indicate standard errors. * indicates 0.01 < *p* < 0.05, ** indicates 0.001 < *p* < 0.01, and *** indicates *p* < 0.001 from paired *t*-test. The inset shows the results of two-way ANOVA on the effect of afforestation (control vs. afforested) and species on soil pH. Interaction indicates afforestation × species

We found a rather mixed result on the post-afforestation change in soil pH (CIP), although on average the change of soil pH across the 549 afforestation−control pairs was not significant (Fig. 2a; mean pH was 6.51 and 6.45 for the control group and the afforestation group, respectively, *p* = 0.076 from a paired *t*-test). Afforested plots had a lower soil pH than corresponding control plots in 53.7% (295/549) of the afforestation−control pairs. In 295 of these plots, the mean and median of H^+^ generation were 44.32 and 6.98 mole ha^−1^, respectively (Supplementary Fig. 1). In contrast, in the remaining 254 (46.3% of the total pairs) pairs, afforested plots had a higher pH value, with mean and median of H^+^ consumption rate of 52.52 and 17.98 mole ha^−1^, respectively (Supplementary Fig. 1). On average, across all the 549 pairs, H^+^ reduced by 3.26 mole ha^−1^ (Supplementary Fig. 1), although insignificant (*p* = 0.69). However, the frequency distribution of soil pH values was significantly altered by afforestation (*p* = 0.02, Siegel−Tukey test). Afforestation reduced the frequencies of both the low (pH < 5) and the high (pH > 7) soil pH values, but increased the frequency of intermediate (5 < pH < 7) pH values (Fig. 2a).

**Fig. 3:**
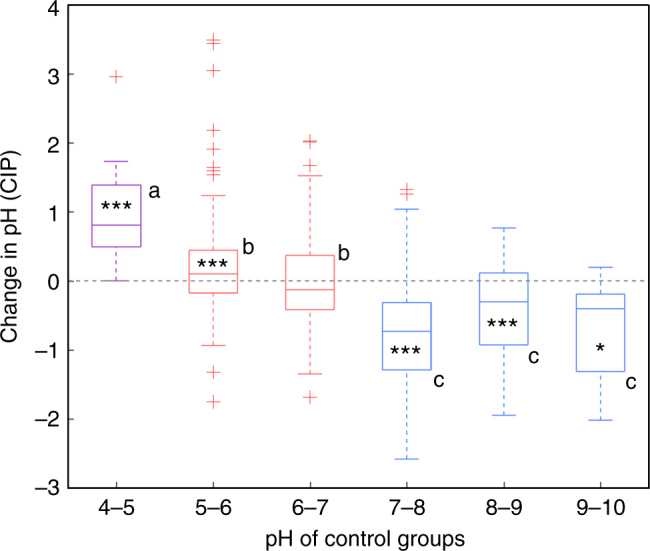
Comparison of CIP across groups of different initial pH. In box-whisker plots, the central mark indicates the median, and the bottom and top edges of the box indicate the 25th and 75th percentiles, respectively. The maximum whisker lengths are specified as 1.5 times the interquartile range and outliers are marked using +. Independent sample *t*-tests with false discovery rate (FDR) correction were conducted to compare data of each group with 0. Symbols *, **, and *** indicate that the null hypothesis could be rejected at a significance level of 0.05, 0.01, and 0.001, respectively. Different letters mean significant differences between different pH groups (*p* < 0.05, one-way ANOVA, post-hoc LSD test)

In the light of these results, we then divided the 549 pairs based on the pH value of control plots, named initial soil pH hereafter, into three groups—pH > 7, 5 < pH < 7, and pH < 5 (Fig. 3, one-way ANOVA, *p* < 0.05)—and investigated the influence of afforestation for each pH group. For the pH > 7 group, afforestation reduced soil pH in 74.1% of the pairs (Supplementary Fig. 2), with an average pH reduction of 0.57 (Fig. 3, *p* < 0.001). This group was further divided into three sub-groups according to initial soil pH, i.e. 7–8, 8–9, and >9. Significant reduction in soil pH was found in all the three sub-groups (*p* < 0.001, *p* < 0.001, and *p* < 0.05, respectively; Fig. 3), and the largest soil pH reduction was found in the sub-group of initial soil pH = 7−8, whose mean value of CIPs was −0.71 (Fig. 3). In contrast, for the pH < 5 group, afforestation increased soil pH in all cases (Supplementary Fig. 2). The mean of CIPs in this group was +0.96 (Fig. 3, *p* < 0.001). For the group 5 < pH < 7, afforestation increased soil pH in about a half (51.7%) of the pairs. The increase in the sub-group 5–6 was modest (mean CIPs is +0.18) but significant (*p* < 0.001). Furthermore, for the group with initial soil pH = 6–7, afforestation-induced soil pH change was not significant (*p* = 0.74).

### Impacts of different tree species on soil pH changes

The impact of afforestation on soil pH was further complicated by the choice of different afforestation species (Fig. 2b). In particular, afforestation with *P. koraiensis* and *P. tabuliformis* significantly reduced soil pH from 5.84 to 5.64 (*p* < 0.05) and from 7.39 to 7.21 (*p* < 0.05), respectively. In contrast, afforestation with *P. sylvestris* var. *mongolica*, *L. gmelinii* and *Populus* spp. had no statistically detectable effect on soil pH (Fig. 2b). Further analyses showed that afforestation species had significant effects on soil pH, but afforestation (control vs. afforested) and the interaction of afforestation and species did not have a significant impact on soil pH (Fig. 2b). Furthermore, the vertical patterns of CIPs also varied among different species. For instance, *P*. *koraiensis* and *P*. *sylvestris* var*.mongolica* significantly acidified the deep layers, while *P*. *tabuliformis* afforestation mainly affected top soil layers (0–20 cm, Supplementary Table 1).

**Fig. 4:**
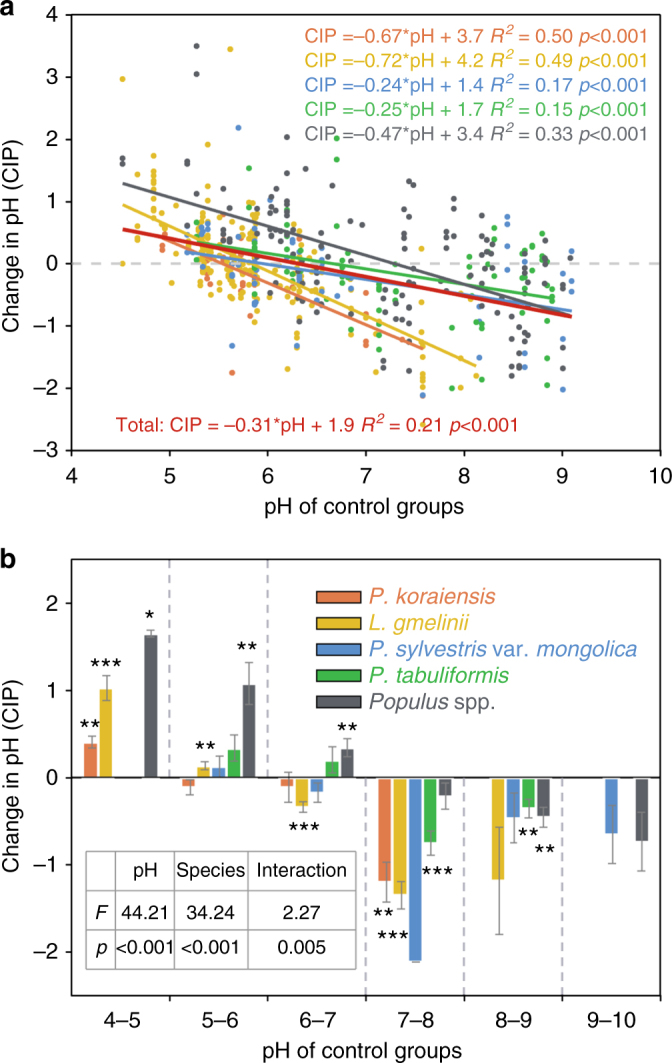
Relationships between change in soil pH and initial pH across five plantation tree species. **a** Plotting of change in soil pH (CIP) against initial soil pH for each of the five plantation species. Solid lines indicate the ordinary least squares (OLS) fit of the linear equation (CIP = a*pH+*b*, where pH indicates the pH of the control group) for each of the five plantation species (the colors are the same as the legend in **b**) and all the species pooled together. **b** The dependence of mean CIP on initial soil pH across the five plantation species. Mean CIP is averaged for each unit of initial soil pH. Error bars indicate standard errors. Independent sample *t*-tests with false discovery rate (FDR) correction were conducted to compare data of each group with 0. Symbols *, **, and *** indicate that the null hypothesis could be rejected at a significance level of 0.05, 0.01, and 0.001, respectively. The inset shows the results of two-way ANOVA on the effect of initial soil pH and species on CIP. Interaction indicates initial soil pH × species

Note that different tree species were planted on different soils, we further used ordinary least squares (OLS) curve fitting to depict the relationship between CIP and initial soil pH for each of the five afforestation species. The results suggested that afforestation decreased soil pH in alkaline soils but increased it in acidic soils consistently across all the five species (Fig. 4). The decrease of CIP with increasing initial soil pH was highest for *L*. *gmelinii* plantations (slope −0.72), followed by that of *P*. *koraiensis* (slope −0.67), *Populus* spp. (slope −0.47), *P*. *tabuliformis* (slope −0.25), and *P*. *sylvestris* var. *mongolica* (slope −0.24); the slope was −0.31 when all the five species were combined (Fig. 4a). We then defined a threshold soil pH (*T*_pH_), which is the point when afforestation-induced CIP changes from positive to negative, shown as the horizontal axis intercept of the OLS curve in Fig. 4a. We found that the overall *T*_pH_ was about 6.3 when all the species were included, with considerably species-specific variations. Specifically, *Populus* spp. plantations had the highest *T*_pH_ (7.3), and the *P*. *koraiensis* plantations had the lowest one (*T*_pH_ = 5.5). Afforestation with *L. gmelinii*, *P*. *sylvestris* var. *mongolica*, and *P*. *tabuliformis* acidified soils when the initial soil pH was >5.8, 5.9, and 6.7, respectively. In general, *Populus* spp. caused the largest increase of soil pH when the initial soil pH < 5.5; but *L*. *gmelinii* and *P*. *koraiensis* resulted in the largest decrease of pH value when the initial pH > 7. Furthermore, result of two-way ANOVA showed that initial soil pH, species, and their interaction all had significant effects on CIP (Fig. 4b).

### Robustness tests of soil pH neutralization by afforestation

The observed neutralization effect of afforestation on soil pH may have also been confounded by some other factors such as original vegetation types, stand age, soil types, climate and net primary productivity (NPP). To test the robustness of our finding, we used generalized linear models (GLM) to evaluate the effects of all the factors (i.e. initial soil pH, afforestation tree species, original vegetation types, stand age, soil types, precipitation, temperature, and NPP) on CIP (Supplementary Table 2). Results confirmed that initial soil pH was the most dominant factor controlling the variation in CIP, followed by afforestation species choices. It is noteworthy that stand age has little impact on CIP variations (Supplementary Fig. 3, Supplementary Table 2), likely due to the wide distribution of sampled sites over a very large region. The spatial heterogeneity of those sites could be large enough to cover up possible effect of stand age on CIP.

Moreover, we investigated the relationship between CIP and initial soil pH across different soil depths (Supplementary Fig. 4), and the results also confirmed the robustness of afforestation-induced soil pH neutralization. For example, similar patterns of CIP were observed at different soil depths, except for *P*. *tabuliformis* soils below 30 cm (*p* = 0.41 at 30–60 cm and 0.64 at 60–100 cm, Supplementary Fig. 4).

## Discussion

Collectively, our results showed that afforestation tended to neutralize soil pH. An earlier meta-analysis study of site-scale observations suggested that afforestation acidified soil globally^14^. Our results, however, suggested that post-afforestation soil pH could change toward either direction (i.e. increased or decreased soil pH), dependent on the initial soil pH value. Afforestation acidified alkaline soils and alkalinized acid soils for all the five afforestation tree species. For a given tree species, there existed a tipping point of soil pH (*T*_pH_), where afforestation changed from increasing to decreasing soil pH. Measuring pre-afforestation initial soil pH and identifying species-specific *T*_pH_ are thus essential for accurately predicting and dealing with post-afforestation soil pH changes.

Afforestation could lead to soil pH neutralization over the long term by altering the balance between soil hydrogen ion generation and consumption during nutrient cycle. Firstly, afforestation-caused changes in litter decomposition and rhizospheric processes^29,30^ may play a major role in soil pH neutralization. The addition of plant residues can increase, decrease or have little effect on soil pH^31–35^, depending on initial soil pH, nitrogen in plant materials, and the proportion of cations and organic anions. The input of plant residues decreases soil pH at high initial soil pH through nitrogen nitrification in the residue^19,20,30^. However, at low initial soil pH, the activity of nitrification bacteria will be suppressed and mineralized nitrogen tends to be ammonified and thus increase soil pH^19,20,31^. In addition, the organic anion-to-acid ratio of plant residues also has a significant role in determining whether litter decomposition would increase or decrease soil pH, subject to the value of initial soil pH as well^31–35^. Moreover, root exudates (e.g. HCO_3_^−^, OH^−^, and H^+^) can modify rhizospheric pH to enhance nutrient uptake by plant roots^36^. Roots often secrete HCO_3_^−^ when plants take up more anions than cations from acid soils, leading to a higher rhizospheric pH than that of bulk soils. By contrast, in calcareous soils, plants take up more cations than anions, which reduces the rhizospheric pH by releasing H^+^ from their roots to maintain charge balance^36^. The differences in the composition of plant residues and rhizospheric processes thus lead to different CIPs across different initial soil pH and species. Furthermore, these processes also vary with soil depths and hence generate the vertical differentiations of CIP.

Secondly, root exudates may also indirectly influence soil pH by solubilizing unavailable soil aluminum (Al), which provides major buffering capacity in acidic soils^37–40^. The soil−root interface of forested soils contains more water-extractable Al than bulk soils, and afforestation may increase soil Al concentrations^41,42^ through rhizospheric processes^43,44^. The ability of hydrated aluminum ions to donate and accept H^+^ would make it eligible to be an acid or a base, depending on pH of soil solution. Under the domination of the reaction Al^3+^  + 2H_2_O <=> AlO_2_^−^ + 4 H^+^, soil pH reaches an equilibrium at about 5−5.5 (the accurate value depends on the total Al concentration^37–39^). When soil pH is below the equilibrium value, Al(OH)_*n*_^3−*n*^ condenses to Al^3+^ through the reaction Al(OH)_3_ (s) + 3H^+^ → Al^3+^ + 3H_2_O, thus reducing [H^+^] and increasing soil pH. Here, Al^3+^ plays the same role as base cations (e.g. Ca^2+^, Mg^2+^) do. When soil pH is above the equilibrium value, Al^3+^ is hydrolyzed to Al(OH)_3_ (s), which releases more H^+^ and thus decreases soil pH. At that time, aluminum ion is an acid cation. However, these equilibrium (or threshold) pH values have only been empirically estimated by few local-scale studies in places such as Sweden^39^ and northeastern United States^45^, which may limit its applications in broader geographical regions without further verifications. In particular, the semi-arid climate in northern China’s forests and their soil properties may differ from that of Skyllberg^39^ and Ross et al.^45^. Therefore, it still remains open questions whether and how Al buffers soil pH in temperate forest of northern China.

Thirdly, afforestation affects soil pH through influencing the base cation cycle^19,20,46–48^. It has been demonstrated that afforestation can decrease soil pH through plant uptake of base cations^19,20^. However, in acid soils, base cations are relatively scanty. Plants therefore need to get cations from deeper soil through hydrological processes^20^. Moreover, the increases of evapotranspiration^48^ caused by afforestation will reduce the leaching loss of base cations^46,47^, and thus increase soil pH. However, we did not observe a consistent relationship between CIP and change in soil moisture content (SMC) (Supplementary Table 3), which may result from the high sensitivity of SMC to weather conditions, for which one-time sampling may not well represent long-term soil hydrological characteristics.

Terrestrial ecosystems are under the threat of soil acidification caused by the deposition of atmospheric nitrogen and sulfur on local^5–7^ and global scales^49^. For instance, bulk nitrogen deposition has increased by approximately 60% in China during the past three decades^50^ and will probably continue to increase in the foreseeable future due to elevated levels of anthropogenic nitrogen inputs to ecosystems^51^. Our study indicates that afforestation has the potential to alleviate soil acidification caused by enhanced acidic deposition with the appropriate selection of tree species and thus could further increase ecosystem productivity and carbon sequestration. Admittedly, further field studies are still needed to determine best tree species according to soil properties, water availability and climate suitability, and designated ecosystem and socioeconomic goals. Possible mechanisms for the observed effect of soil pH neutralization by afforestation and its potential in mitigating soil acidification caused by increased acidic deposition also remain to be investigated, both qualitatively and quantitatively. Nonetheless, our finding challenges the conventional notion that afforestation usually acidifies soils. Instead, our comparative study along a 2000 km transection in northern China finds that afforestation neutralizes soil pH. This can be another benefit of afforestation: when appropriate tree species are selected based on initial soil pH, afforestation may have the potential to modify soil pH, which will promote soil health and increase ecosystem productivity^12^.

## Methods

### Study region

Soils samples were collected from northern China, in the provinces of Heilongjiang, Jilin, Liaoning, Hebei, Shanxi, Shaanxi, and the Inner Mongolia Autonomous Region. The sampling region covered most of the TNSDP area, which extends from 34.20 to 51.80°N and 106.81 to 133.31°E (Fig. 1). Mean annual temperature and precipitation range from −3 to 15 °C and 355 to 1068 mm year^−1^, respectively. Dominant soil types in this region include black soil, bog soil, brown coniferous forest soil, brown earths, brown pedocals, castanozems, chernozems, cold brown calcic soil, yellow earths and yellow-brown earths^52^, roughly corresponding to phaeozems, gleysols, humic cambisols, haplic/albic luvisols or eutric/dystric cambisols, haplic calcisols, kastanozems, chernozems, cambisols, haplic alisols, and ferric/haplic luvisols of the United Nations Food and Agriculture Organization (FAO)^53^, respectively.

### Sampling design

We established a paired afforestation-control system to evaluate the effects of afforestation on soil pH. For each site, we chose one non-afforested plot as a control plot and several afforested plots with different stand ages (in some sites with only one tree species and one stand age, we sampled only one afforested plot; in other sites, we chose at least two afforested plots to cover different tree species and stand ages (maximum = 30 afforested plots)). Within a site, the distance between any afforested plot to its corresponding non-afforested control plot was less than 2.5 km to minimize the variation in soil and climatic properties between the pair; and that between any two afforested plots was more than 50 m but less than 5 km. It is noteworthy that the pre-afforestation vegetation types and soil types of afforested plots were the same as that of the corresponding control plots, according to the records provided by local forestry administrations. Specifically, the original vegetation types in this study include cropland, barren land, grassland, natural forest, and desert. Data of stand afforestation age were also obtained from local forestry administrations. For each plot, we dug three replicate soil profiles to a depth of 1 m. For each profile, soils were sampled from six layers (0–5, 5–10, 10–20, 20–30, 30–60, and 60–100 cm) using a cutting ring. Therefore, except for a few plots that we could not reach to 1 m in depth, we collected 18 soil samples in each plot; and 11,118 soil samples for the whole project. We also recorded the planted tree species for each afforested plot; and as a result, five major afforestation tree species, including *P*. *koraiensis*, *L*. *gmelinii*, *P*. *sylvestris* var. *mongolica*, *P. tabuliformis*, and *Populus* spp. (including *Populus simonii*, *Populus* × *beijingensis*, and *Populus* × *xiaohei*), were documented in this study.

### Laboratory measurement of soil pH

All soil samples were air-dried to constant weights in a ventilated room, and roots and stones were removed. Samples were then gently grinded in a mortar and passed through 2-mm sieves. The pH of each sample was measured in 1:2.5 mixtures of soil and deionized water with a pH meter (PHS-3C, Lei-ci). Soil solutions were shaken for 30 min and then kept static for 5 min before pH measurement.

### Data analysis

Because each plot included three replicate profiles and each profile included samples from different soil depths, we needed to appropriately derive the value of soil pH for each plot so that it could represent the mean concentration of hydrogen ions of soil in the whole plot. The mean concentration of hydrogen ions (*H*_p_) for the entire soil profile was calculated from hydrogen ion concentration, [H^+^], of each layer weighted by its thickness:

1 $$H_{\rm p} = \frac{{\mathop {\sum }\nolimits_{{{j = 1}}}^6 H_j^\ast w_j}}{{\mathop {\sum }\nolimits_{{{j = 1}}}^6 w_j}},$$

where *w*_*j*_ and *H*_*j*_ are the thickness and the concentration of hydrogen ions of the *j*th layer, respectively. Similarly, the mean concentration of hydrogen ions in a plot ($\overline {H_{\rm p}}$) was calculated by averaging *H*_p_ of its three replicate profiles, then we calculated the total hydrogen ions of a plot and transformed it into the hydrogen ions in one-hectare soil (mole  ha^−1^). However, the hydrogen ion content varies across some orders of magnitude and it is not approximately normal distribution, so logarithmic transformation is needed. Therefore, we got the average pH for each plot from a log transformation of $\overline {H_{\rm p}}$:

2 $${\mathrm{pH}}_{{\mathrm{plot}}}{\mathrm{ = - log}}_{{\mathrm{10}}}^{\bar H_{\rm P}}.$$

For each afforestation−control pair, we calculated the change in pH (CIP) as:

3 $${\mathrm{CIP = pH}}\;{\mathrm{in}}\;{\mathrm{afforested}}\;{\mathrm{plot}}\;{\mathrm{-}}\;{\mathrm{pH}}\;{\mathrm{in}}\;{\mathrm{its}}\;{\mathrm{corresponding}}\;{\mathrm{control}}\;{\mathrm{plot}}.$$

Note that pH is the negative logarithm of [H^+^] and thus CIP represents the effected changes in the ratio of [H^+^] caused by afforestation. Given that each afforested plot corresponded to one control plot, we got 549 pairs of control-afforested data to conduct the following analyses. Firstly, we applied a Siegel−Tukey test to compare the frequency distributions of soil pH between the control and afforested groups. Secondly, we used a paired *t*-test to evaluate the difference of soil pH between the paired plots. In the same time, we also conducted independent sample *t*-tests to find if CIPs were significantly different from 0. False discovery rate (FDR) correction^54^ was used to control potential error rates in multiple comparisons. FDR correction sorts all the *p* values of *t*-tests in ascending order: *p* (1) <= *p* (2)⋯ <= *p* (m), and compares each *p* value (*p*(*i*)) with q*i/*m* (where *i* is the order, *m* is the number of groups, *q* is pre-defined significance level, e.g., 0.05, 0.01, and 0.001). If *p*(*i*) <= q*i/*m*, the null hypothesis is rejected. Thirdly, we used one-way and two-way analysis of variance (ANOVA) to test the effect of different factors on soil pH and CIP. Those factors used in this analysis included afforestation (control vs. afforested), tree species, and initial soil pH. Fourthly, we performed ordinary least square (OLS) to examine the correlation between CIP and initial pH. Finally, we conducted GLM to synthetically evaluate the effects of all the factors (i.e. initial soil pH, tree species, original vegetation types, stand age, soil types, precipitation, temperature, and NPP) on CIP (Supplementary Table 2). Data of mean annual precipitation and temperature were acquired from the China Meteorological Forcing Dataset^55,56^. This data set was created by merging a variety of data sources and included 3-h data at a resolution of 0.1° x 0.1°. For NPP, we used the Moderate Resolution Imaging Spectroradiometer (MODIS) data, MOD17A3 data set^57^. Data of soil type were obtained from the Harmonized World Soil Database (HWSD) v.1.2^58^. Longitude and latitude of each plot recorded by global positioning system were used to extract the data of climate and NPP for each plot. Furthermore, partial regressions were used to evaluate the effects of stand age on CIP after controlling for initial soil pH (Supplementary Fig. 3). Changes in soil moisture content (SMC) were calculated from SMC in afforested plots minus SMC in control plots (Supplementary Table 3). Independent *t*-tests were used to exam whether the changes were significantly different from 0. All statistical analyses were conducted using MATLAB R2012b (MathWorks, Natick, MA, USA).

### Data availability

The authors declare that the source data supporting the finding of this study are provided with the paper.

## Electronic supplementary material

Supplementary Information
