# Supplementary material for: Afforestation neutralizes soil pH
Source: Nat Commun. 2018 Feb 6;9:520. doi: 10.1038/s41467-018-02970-1 (PMC5802719; doi:10.1038/s41467-018-02970-1)
Supplement: Supplementary file 1 — Supplementary Information [file 41467_2018_2970_MOESM1_ESM.pdf]

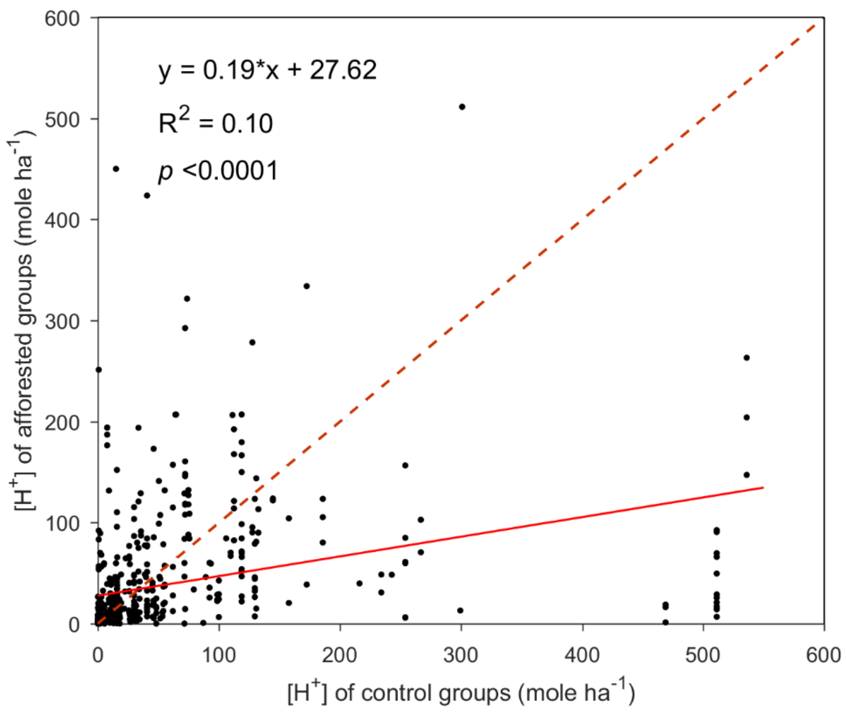

Supplementary Figure 1 Relationship between [H^+] in control and afforested plots. The dashed line is the 1:1 line and the solid line indicates the OLS fit between [H^+] in afforested and control plots. Three outliers (3727, 1266, 747) of the y-axis are not shown in the graph.

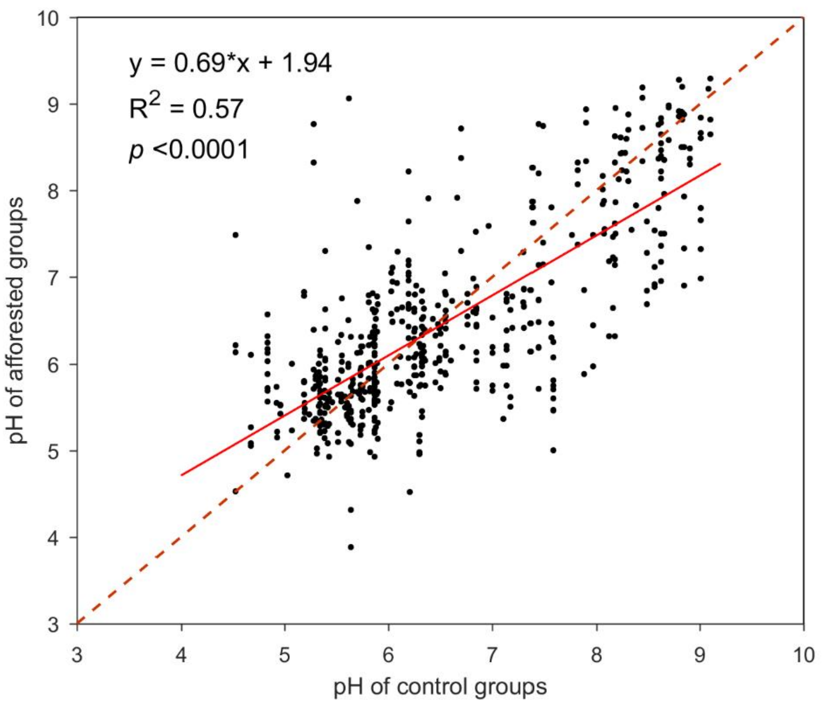

Supplementary Figure 2 Relationship between control and afforested soil pH groups. The dashed line is the 1:1 line and the solid line indicate the OLS fit between pH values in afforested and control groups.

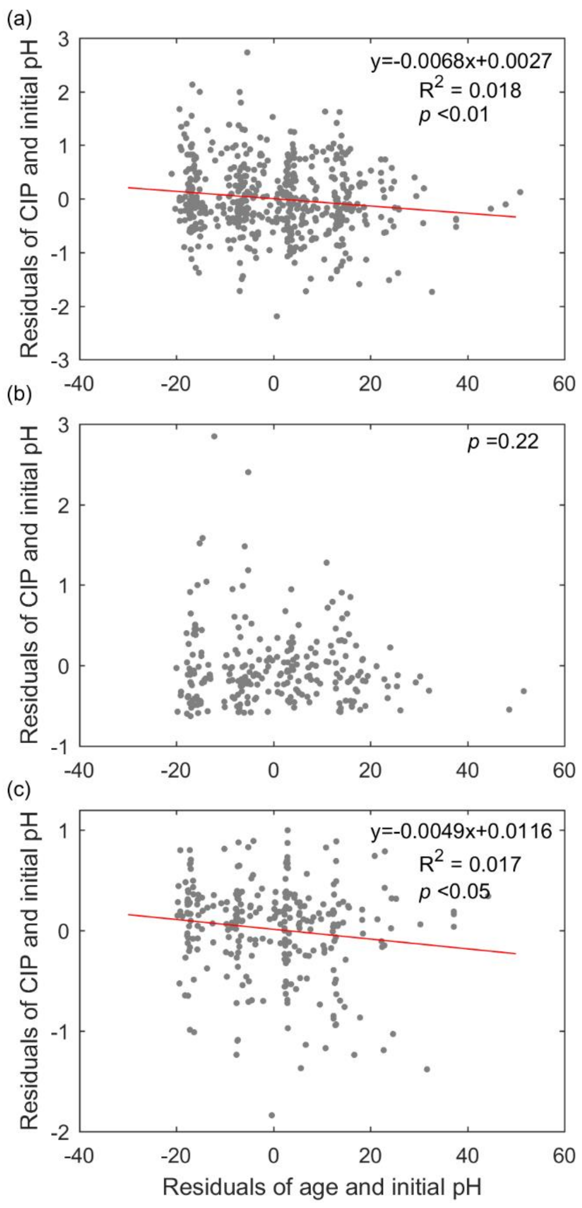

Supplementary Figure 3 The relative effect of stand age on CIP. The red lines are from partial linear regressions. Only significant fitted lines are shown in the graph. Partial linear regressions are conducted by all the data (a), positive CIP (b), and negative CIP (c), respectively.

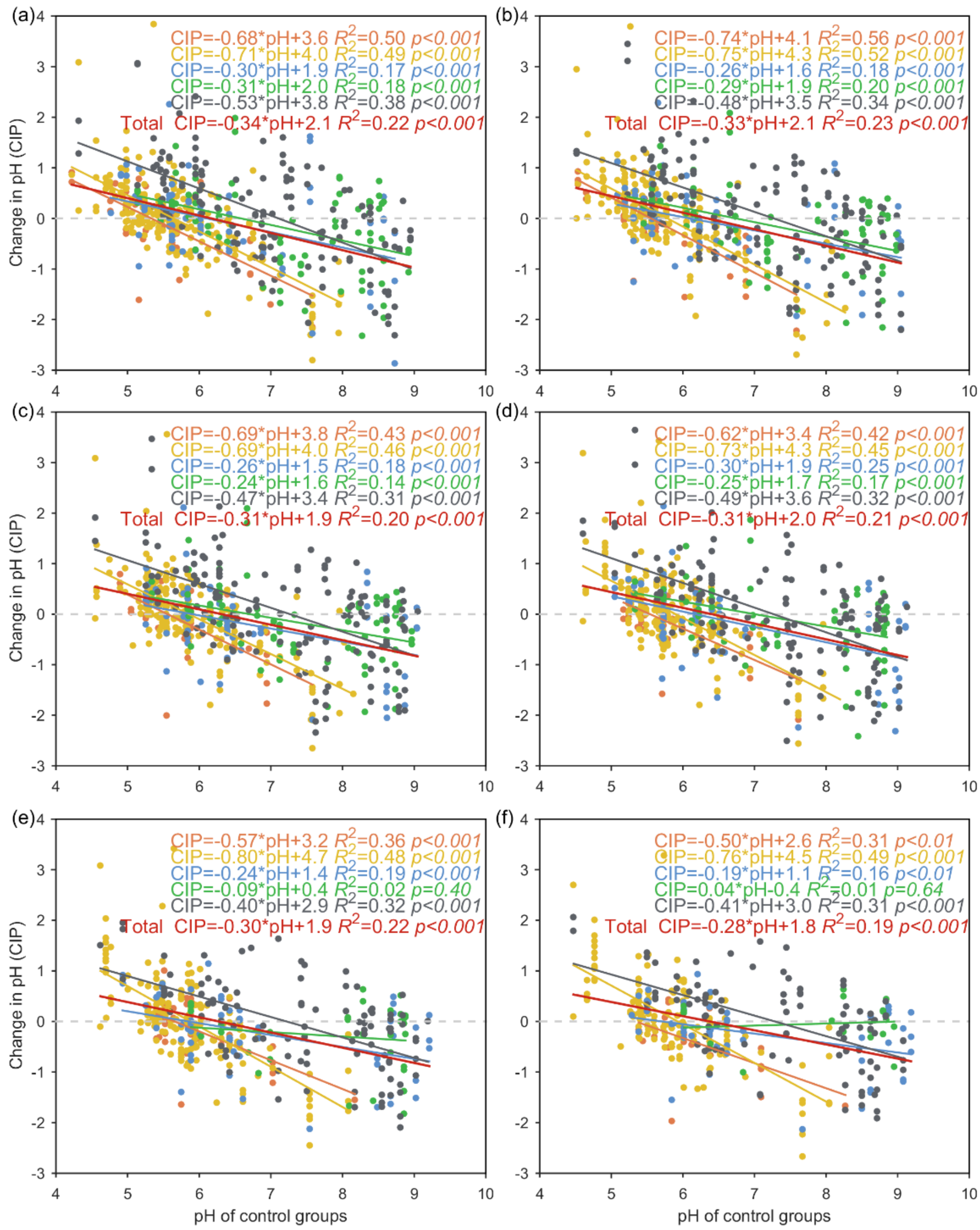

Supplementary Figure 4 Distribution of change in soil pH (CIP) from initial pH across the five tree species in soil with different depths. (a), (b), (c), (d), (e) and (f) were analyzed for soil with different depths (0-5 cm, 5-10 cm, 10-20 cm, 20-30 cm, 30-60 cm, and 60-100cm, respectively). The solid lines in each panel indicate the OLS fit of the linear equations for the five tree species and their summation, and the colors are accordant with Figure 4a.

Supplementary Table 1 Change in soil pH (CIP) across five tree species in soil with different depths

	Pinus koraiensis	*Larix gmelinii*	*Pinus sylvestris* var. *mongolica*	*pinus tabuliformis*	*Populus* spp.
0-5 cm	-0.12	-0.04	-0.07	-0.19*	0.08
5-10 cm	-0.07	0.01	-0.11	-0.17*	0.08
10-20 cm	-0.19	-0.02	-0.19*	-0.17*	0.03
20-30 cm	-0.21*	0.03	-0.15	-0.12	-0.01
30-60 cm	-0.19*	0.02	-0.22*	-0.29*	-0.06
60-100 cm	-0.42**	0.02	-0.24**	-0.04	-0.02

* indicates $0.01<p<0.05$, ** indicates $0.001<p<0.01$, and *** indicates $p<0.001$ in single sample t-test, comparing with 0.

Supplementary Table 2 Cumulative adjusted R^2 for generalized linear model (GLM) selected by forward-selection method

Factors	Cumulative adjusted R^2(%)
Initial pH	21.6
Tree species	31.0
Initial pH × Tree species	35.8
Precipitation	39.1
Soil type	41.5
Initial pH × Soil type	43.5
Stand age	46.4
Soil type × Precipitation	48.6

Factors including initial pH, tree species, stand age, soil types, original vegetation type, precipitation, temperature, NPP were put into the model, but only factors which marginal contribution to cumulative adjusted R^2 more than 1% were showed in the table.

Supplementary Table 3 Changes in soil moisture content (%) across five tree species in soils with different depths.

			All plots		
	Pinus koraiensis	*Larix gmelinii*	*Pinus sylvestris* var. *mongolica*	*Pinus tabuliformis*	*Populus* spp.
0-5 cm	-2.11	-2.05	-9.45 ***	0.78	-5.33 *
5-10 cm	-3.69	0.87	-7.45 ***	-0.37	-7.38 **
10-20 cm	-4.18 *	-2.06	-6.41 ***	-0.24	-5.87 *
20-30 cm	-0.55	0.74	-0.71	-0.33	-5.66 **
30-60 cm	-2.85 *	-1.58	-1.69 *	-0.51	-2.58
60-100 cm	0.67	-1.79	0.27	-1.06	-3.98
			pH increased plots only		
	Pinus koraiensis	*Larix gmelinii*	*Pinus sylvestris* var. *mongolica*	*Pinus tabuliformis*	*Populus* spp.
0-5 cm	2.90	-7.35 *	-4.37	-4.65	-8.32
5-10 cm	-0.40	-1.61	-2.88	-4.17 *	-13.19 **
10-20 cm	0.03	-4.74	-3.50	-3.90	-10.71 *
20-30 cm	3.61	-1.54	1.01	-1.63	-7.34 *
30-60 cm	-2.25	-2.36	-1.14	-1.00	-3.80
60-100 cm	0.58	-4.80	-0.10	-0.73	-6.93

* indicates $0.01<p<0.05$, ** indicates $0.001<p<0.01$, and *** indicates $p<0.001$ in independent sample t-test, comparing with 0.
